# Cold Acclimation for Enhancing the Cold Tolerance of Zebrafish Cells

**DOI:** 10.3389/fphys.2021.813451

**Published:** 2022-01-28

**Authors:** Huamin Wang, Ying Wang, Minghui Niu, Linghong Hu, Liangbiao Chen

**Affiliations:** ^1^International Research Center for Marine Biosciences, Ministry of Science and Technology, Shanghai Ocean University, Shanghai, China; ^2^Key Laboratory of Exploration and Utilization of Aquatic Genetic Resources, Ministry of Education, Shanghai Ocean University, Shanghai, China; ^3^Shanghai Collaborative Innovation for Aquatic Animal Genetics and Breeding, Shanghai Ocean University, Shanghai, China

**Keywords:** ZF4 cells, cold stress, cold acclimation, oxidative stress, apoptosis

## Abstract

Cold stress is an important threat in the life history of fish. However, current research on the tolerance mechanisms of fish to cold stress is incomplete. To explore the relevant molecular mechanisms enabling cold stress tolerance in fish, here we studied ZF4 cells subjected to short-term (4 days) low temperature stress and long-term (3 months) low temperature acclimation. The results showed that cell viability decreased and the cytoskeleton shrank under short-term (4 days) low temperature stress, while the cell viability and the cytoskeleton became normal after cold acclimation at 18°C for 3 months. Further, when the cells were transferred to the lower temperature (13°C), the survival rate was higher in the acclimated than non-acclimated group. By investigating the oxidative stress pathway, we found that the ROS (reactive oxygen species) content increased under short-term (4 days) cold stress, coupled with changes in glutathione (GSH), catalase (CAT), superoxide dismutase (SOD) enzyme activity levels. In addition, overproduction of ROS disrupted physiological cellular homeostasis that generated apoptosis *via* the activation of the mitochondrial pathway. However, when compared with the non-domesticated group, both ROS levels and apoptosis were lowered in the long-term (3 months) domesticated cells. Taken together, these findings suggest that cold acclimation can improve the low temperature tolerance of the cells. This exploration of the mechanism by which zebrafish cells tolerate cold stress, thus contributes to laying the foundation for future study of the molecular mechanism of cold adaptation in fish.

## Introduction

As the major component of aquatic fauna, fish species are often exposed to wide fluctuations of water temperature, which plays an essential role in their growth, survival, and reproduction (Vondracek et al., 1988; Schmidt and Starck, 2010; Shahjahan et al., 2017). Most vital activities decline or halt when fish are exposed to lower water temperatures, such as for zebrafish (*Danio rerio*), whose embryos’ development slows down as the temperature drops (Kimmel et al., 1995; Schmidt and Starck, 2010). Furthermore, low temperature stress can cause gill apoptosis in both zebrafish and tilapia (Hu et al., 2016). Studies show that when exposed to low temperature, the metabolic rate decreased and energy homeostasis were disrupted in orange-spotted grouper (*Epinephelus coioides*) and coho salmon (*Oncorhynchus kisutch*) (Larsen et al., 2001; Sun et al., 2019). Even worse, in aquaculture and natural waters, a substantial temperature decline may trigger the death of various fish (Beitinger et al., 2000; Liang et al., 2015). Therefore, it is necessary to investigate how fish physiologically respond to low temperature, and in recent years, many studies have examined fish responses to cold stress. For example, fish can tolerate cold stress by adjusting their metabolism, which includes increased energy demand and glycolysis and amino acid catabolism (Islam et al., 2021; Schleger et al., 2021). Furthermore, fish can also alter the rate of protein synthesis and the activity of enzymes under low temperature (Ren et al., 2021; Yilmaz et al., 2021). Nevertheless, those extensive research activities have focused on short-term stress responses to cold, leaving little known about the specific molecular mechanism by which fish improve their cold tolerance during the long-term process of acclimatization. Recently, many studies have shown that cold acclimation can enable the physiological activities of fish to adapt to a lower temperature (Johnson et al., 2014; Klaiman et al., 2014; Keen et al., 2017). However, research investigating the role of long-term temperature acclimation in specific mechanisms that enhance the low temperature tolerance of fish remains scarce.

Cold stress can cause oxidative stress by inducing the production of excess reactive oxygen species (ROS). When levels of active oxygen increases in an organism, the system of antioxidant enzymes that can eliminate ROS are activated in the body (Ali et al., 2010). For example, lipid and protein peroxidation products of the northern moray eel (*Zoarces viviparus*) increased markedly after its acute exposure to low temperature (Heise et al., 2006). The expression of antioxidant enzyme genes of tilapia (*Oreochromis niloticus*) and pufferfish (*Takifugu obscurus*) also increased considerably under low temperature stress (Cheng et al., 2017; Yilmaz et al., 2021). In pufferfish (*Takifugu obscurus*) and euryhaline milkfish (*Chanos chanos*), cold stress induced oxidative stress in their blood and hepatocytes, respectively (Cheng et al., 2018; Chang et al., 2021). However, excessive oxidative stress may trigger oxidative damage to organisms, which is likely to directly result in macromolecular damage and cell apoptosis (Xing et al., 2016; Zhuang et al., 2017). Recent reports have shown that many fish apoptosis-related genes are significantly induced by low temperature (Heise et al., 2006; Sun et al., 2019). Therefore, overcoming oxidative stress is also a great challenge for fish under conditions of hypothermic stress.

Zebrafish is a key model in zoology, being widely used in developmental biology, genetics, physiology, toxicology, and other fields of animal research (Grunwald and Eisen, 2002; Ruzicka et al., 2019). We should note that zebrafish is tropical bony fishes capable of tolerating water temperatures ranging from 10.6°C ± 0.5°C to 41.7°C ± 0.3°C (Cheryl and Beitinger, 2005). Hence, zebrafish is widely used in the study of fish temperature stress (López-Olmeda and Sánchez-Vázquez, 2011). Much like its individual fish, the embryonic fibroblast cell line of zebrafish (ZF4) has an optimum growth temperature of 28°C and exhibits a wide range of temperature tolerance. Accordingly, ZF4 cells are considered a suitable cell model for the low temperature stress research, and were selected for study here. To maintain cellular homeostasis, fish must activate a physiological cascade of acclimation strategies while under cold stress (Donaldson et al., 2008; Tseng et al., 2011). To better explore those responses, the effects of short-term cold stress and long-term cold acclimation on zebrafish cell physiology, oxidative stress, and apoptosis should be investigated. This study not only provides ideas toward low temperature stress but also deep insights into the theory that cold acclimation can improve low temperature tolerance.

## Materials and Methods

### Cell Culture and Treatment

ZF4 cell lines were purchased from the American Type Culture Collection (ATCC, Cat No. CRL 2050). These cells were thawed, resuscitated, and passaged at 28°C, 5% CO_2_, in Dulbecco’s modified Eagle’s medium/F12 nutrient mix (SH30023.01B, Hyclone, Thermo Scientific, MA, United States) with 10% fetal bovine serum (FBS) and 1% penicillin-streptomycin-glutamine solution (SV30082.01, Hyclone, Thermo Scientific, MA, United States). The temperature treatment for the cold stress experiment was selected to be consistent with previous reports on zebrafish (Wu et al., 2011; Han et al., 2016). For the short-term cold stress group, when adherent cells had reached 80–90% confluency, the cells were moved to 18 and 13°C incubators with 5% CO_2_ for 4 days. For the cold acclimation group, the culture media was changed every 2–3 days and the cells were passaged regularly at 18°C, with 5% CO_2_, for 3 months. After undergoing the cold acclimation treatment, the low temperature domesticated cells were divided into 13°C incubators with 5% CO_2_ for a 5 day period. Meanwhile, the cells at 28°C were transferred into 13°C incubators, also for 5 days, to serve as the control group. The morphology of these treated cells was observed and photographed daily under a fluorescent inverted microscope.

### Cell Viability Assay

Cell viability was detected using a CCK-8 assay kit (C0038, Beyotime Biotechnology, Shanghai China) and following the manufacturer’s instructions. The ZF4 cells were seeded onto a 96-well plate, at a density of 5000 cells per well, with 100 μL of medium. After the cold temperature treatment, 10 μL of the CCK-8 reagent was added to each well at various time points, and then all cells were further incubated for 4 h. Their absorbance at 450 nm was quantified with an enzyme-linked immunosorbent assay reader.

### Phalloidin Staining and Inverted Fluorescence Microscope

The cells that climbed onto the carry sheet glass (20 mm) were cultured in an incubator. At the start of the experiment, the cells were washed three times with 1 × PBS. Then, the cells were fixed with 4% paraformaldehyde for 15 min at room temperature, and then washed again with 1 × PBS. After fixation, cells were incubated in a 0.5% Triton X-100 solution for 5 min, and then washed again with 1 × PBS. The cytoskeleton was stained with TRITC-conjugated phalloidin (CA1610, Solarbio, Beijing, China) for 30 min, away from any light, at room temperature. Next, the cells were washed three times with 1 × PBS. Finally, DAPI (100 nM in PBS, Invitrogen, CA, United States) was used to counterstain the nuclei at room temperature for 40 s. Their fluorescence images were captured under a confocal microscope (Leica, SP8 FALCON, Germany).

### Antioxidant Enzyme Activity

ZF4 cells were seeded in 100-mm-diameter dishes (2 × 10^5^ cells per dish) and cultured to 90% confluency. Their cold stress treatment was performed as described above. Then all the cells were collected and washed with 1 × PBS two times and centrifuged for a final collection. The respective activity of superoxide dismutase (SOD), catalase (CAT), and glutathione (GSH) enzymes was determined using commercial assay kits (Jiancheng Bioengineering Institute, Nanjing, China). The activities of SOD, CAT and GSH are expressed in U/mg protein.

### Quantification of Reactive Oxygen Species

To monitor the intracellular generation of ROS, viable ZF4 cells were collected for staining. The generation of intracellular ROS was detected using 2′,7′-Dichlorodihydrofluorescein diacetate (DCFH-DA, D6883, Sigma, MO, United States) as the peroxide-sensitive fluorescent probe. The cell suspension (5 × 10^4^ cells/mL) was incubated with 10 μM DCFH-DA for 30 min in the absence of any light. After being washed with 1 × PBS, the cell suspension was analyzed by flow cytometry within 30 min (BD FACS Accuri C6; BD Biosciences, CA, United States). Lastly, data analysis of intracellular ROS was conducted using FlowJo 10 software.

### Mitochondrial Membrane Potential Assay

Two assays were used to evaluate the effects on cells’ mitochondrial membrane potential. Mitochondrial membrane potential (MMP) was analyzed using JC-1 (T4069, Sigma, MO, United States) in both flow cytometric and cell fluorescence assays. All cells were washed with ice-cold 1 × PBS and stained with 5 mg/mL JC-1 for 30 min in the dark. Then, each cell suspension was quantified by flow cytometry (BD FACS Accuri C6; BD Biosciences, MO, United States). Fluorescence images were acquired using a confocal microscope (Leica, SP8 FALCON, Germany).

### Apoptosis Assay

According to the manufacturer’s instructions, cell apoptosis was detected by Annexin V-FITC/PI Apoptosis kit (CA1020, Solarbio, Beijing, China). The cells were dual-stained with Annexin V-FITC and PI at room temperature for 20 min in the dark. This method simultaneously determines the percentage of live and total apoptotic cells (early and late apoptotic). After incubation and washing, the cell suspension was analyzed by flow cytometry (BD FACS Accuri C6; BD Biosciences, MO, United States). Data analysis was performed using FlowJo 10 software.

### Statistical Analysis

Results of each experiment were obtained three times (*n* = 3 replicates), with three biological replicates used in every experiment, unless noted otherwise. The experimental data are expressed here are the mean ± standard deviation (SD). All data analyses and graphing were done using IBM SPSS Statistics v20 and GraphPad Prism 8.0. The results were analyzed by single-factor analysis of variance (one-way ANOVA) and by the paired *t*-test. A result of *P* < 0.05 was considered significant (*); that of *P* < 0.01 was considered extremely significant (**); that of *P* < 0.001 was considered extremely significant (***).

## Results

### Cold Acclimation Affected Cell Morphology and Viability

To investigate whether cold acclimation promotes cold tolerance, short-term cold stress (4 days) and long-term cold acclimation treatments (3 months) were applied to ZF4 cells. After being treated at 18°C for 4 days, the cells displayed a slight contraction and widening of the cell gaps. When the treatment temperature dropped from 18 to 13°C and was maintained for 4 days, the cells were observed to shrink and grow into strips, with the gaps between cells becoming larger and the boundary of cells unclear. In addition, some dead cells were observed floating in the culture medium. However, when we exposed the cells to 18°C for 3 months of cold acclimation, that contraction of cells and those cell gaps were not discernible. Next, we exposed the cold acclimation group (18°C for 3 months) and the non-acclimation group (28°C) to 13°C for 5 days, which revealed the shedding of cells was mild to moderate in the acclimation group but severe in the non-acclimation group.

**FIGURE 1:**
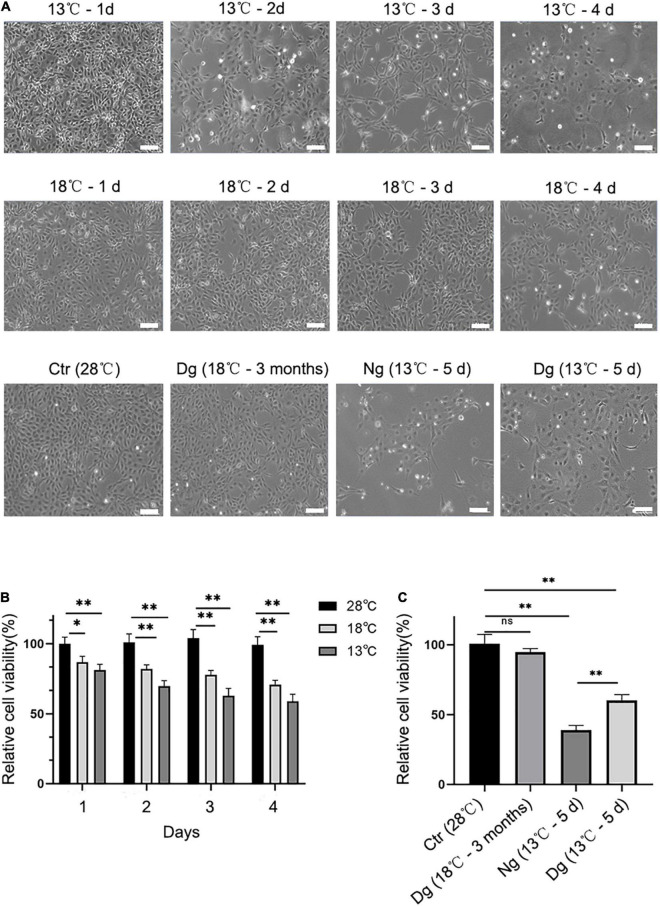
Effect of cold stress on cell morphology and cell viability. Cells were allowed to grow in the incubator at 28°C in 5% CO2 and then treated with low temperature (13 and 18°C). Ctr, the control group; Dg, the domesticated group; Ng, the non-domesticated group. **(A)** Morphological pictures of ZF4 cells under normal or low temperature conditions. Cell morphology was examined under an inverted microscope at a 20 × magnification. Scale bar = 100 μm. **(B,C)** The CKK-8 assay was used to measure the viability of groups of short-term low temperature stress and long-term low temperature acclimation cells. The experiments were repeated three times. The results are presented as the mean ± SD (*n* = 6); *P < 0.05, **P < 0.01.

Only a few cells were observed in the non-domesticated group, and the cell margin appeared fairly blurry (Figure 1A). In parallel, we used the CCK8 assay to detect cell viability in both the cold stress and cold acclimation groups. These results indicated no significant difference in the cell viability versus the control group (28°C) (Figure 1B). In stark contrast, the cell viability decreased dramatically in both cold stress groups (*P* < 0.05 vs. control). Further, after transferring the cells to a 13°C exposure, a significant difference was observed when comparing the cell viability of the cold acclimation group (18°C for 3 months) with the control group (*P* < 0.05) (Figure 1C). All these results suggested that cold acclimation enables the cell to remain intact and viable under lower temperatures. Given that cell viability was reduced by ∼50% in going from normal conditions (28°C) to 13°C exposure for 4 days, in subsequent experiments these variables were selected as the standard time points for the induction of ZF4 cell apoptosis.

### Effects of Cold Stress on the Cytoskeleton Structure of ZF4

**FIGURE 2:**
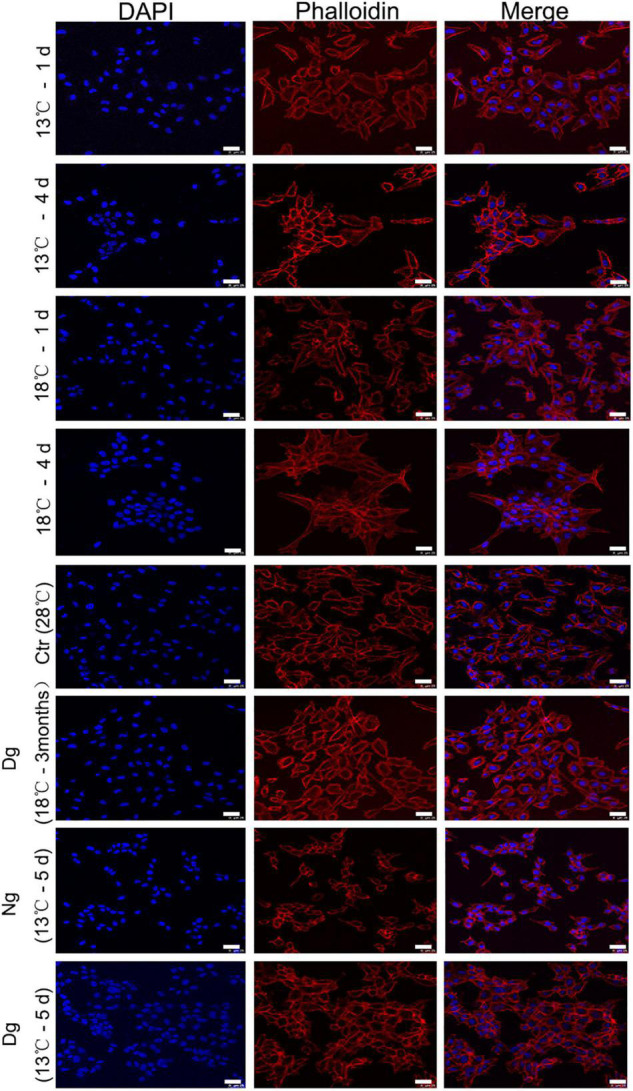
Low temperature acclimation helps to maintain the ZF4 cell cytoskeleton. F-actin was labeled with rhodamin-phalloidin following exposure to low temperatures at different time points in ZF4 cells. The cell cytoskeletons were stained with phalloidin (red) and the staining of nuclei was performed with DAPI (blue). Laser scanning confocal microscopy was used to visualize the cytoskeleton of the cell. Scale bar = 25 μm. Ctr, the control group; Dg, the domesticated group; Ng, the non-domesticated group.

To observe the cell cytoskeleton under cold stress conditions, phalloidin-strained F-actin was used to stain the cytoskeleton. This F-actin cytoskeleton was then detected by rhodamine-phalloidin (red) staining, while the nuclei were visualized with DAPI (blue). As seen in Figure 2, the control group’s (28°C) cells were arranged regularly and well clustered. However, the morphology of ZF4 cells underwent gradual shrinkage in the two cold stress groups, and became rounder with decreasing temperature. After a 3-month period of cold acclimation, the cell morphology gradually became regular in appearance. Yet, when we transferred the cells to the lower temperature without acclimation, the F-actin distribution lost its regularity. Furthermore, a large amount of granular fluorescence was found aggregating into clusters. Collectively, these results illustrated that cold stress significantly affected the cytoskeleton, but cold acclimation can protect the cells from death by stabilizing the cytoskeleton structure.

### Cold Acclimation Inhibits Cold Stress-Induced Oxidant Stress in ZF4 Cells

**FIGURE 3:**
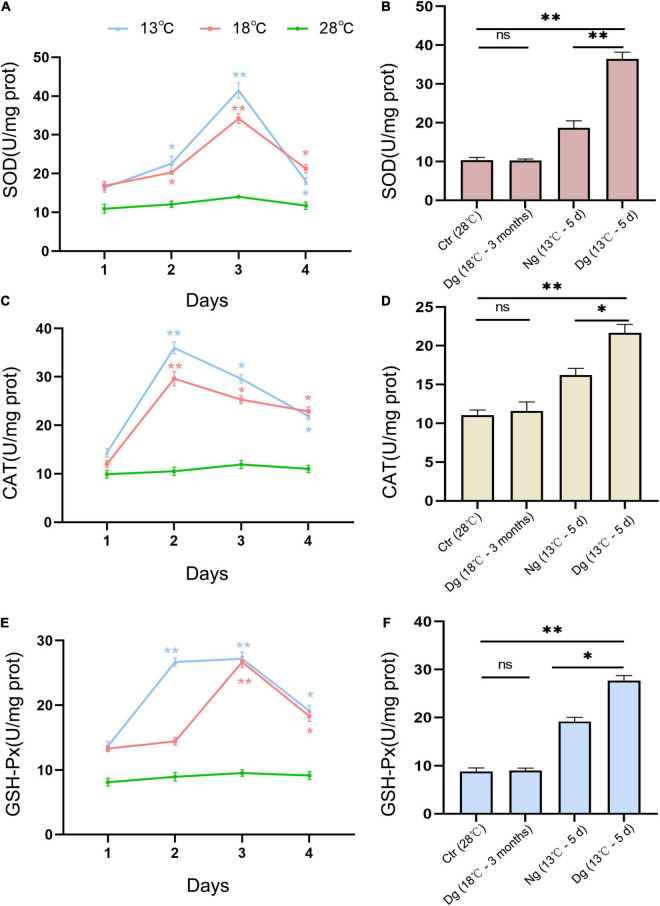
Effects of cold stress on the antioxidant capacity of ZF4 cells. The effects of short-term low temperature stress and long-term cold acclimation on the antioxidant activities of SOD **(A,B)**, CAT **(C,D)**, and GSH-Px **(E,F)** in the ZF4 cells were measured using the corresponding detection kits. Ctr, the control group; Dg, the domesticated group; Ng, the non-domesticated group. The experiments were repeated three times. The results are presented as the mean ± SD (*n* = 3); *P < 0.05, **P < 0.01.

To estimate the endogenous anti-oxidative capacity and oxidative stress status of ZF4 cells, we measured several antioxidant enzyme activities (SOD, CAT, and GSH) and ROS. Such antioxidant enzymes are considered the primary defense system against oxidative stress in cells. As Figure 3 shows, SOD, CAT, and GSH activities were increased in a similar manner in both cold stress groups as the temperature decreased. CAT activity increased sharply on the 2nd day, while SOD and GSH activities peaked on the 3rd day (*P* < 0.01). After 3 months of acclimation, these cell enzyme activity levels had decreased substantially to below those of the control group (Figures 3B,D,F). At a lower temperature of 13°C, the antioxidant enzymes of the acclimated group were significantly higher (*P* < 0.05) in comparison with the non-acclimated group.

**FIGURE 4:**
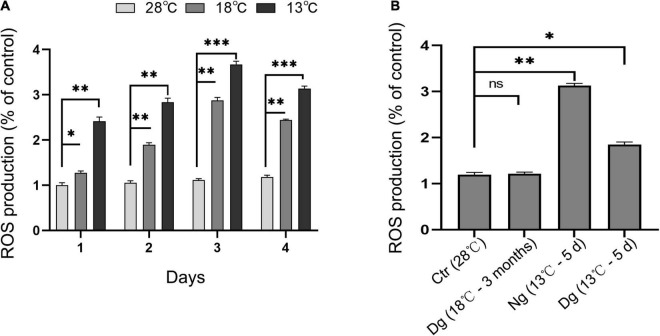
Cold acclimation suppressed cold-induced oxidant stress in ZF4 cells. Ctr, the control group; Dg, the domesticated group; Ng, the non-domesticated group. **(A,B)** Cellular ROS production under cold stress. ROS was detected by flow cytometry and analyzed using FlowJo software. The results are presented as the mean ± SD (*n* = 3); *P < 0.05, **P < 0.01, ***P < 0.001.

Reactive oxygen species is an important indicator of cellular oxidant stress induced by cold stress. As shown in Figure 4, ROS increased significantly in both cold stress groups at each time point vis-à-vis the control group (*P* < 0.05); however, the original ROS content was restored after cold acclimation (*P* > 0.05). When the cells were transferred to the lower temperature exposure, the ROS levels were lower in acclimated than non-acclimated group (*P* < 0.01). All these results indicated that cold acclimation protected the ZF4 cells against the oxidative stress induced by cold stress.

### Effects of Cold Stress on Mitochondrial Membrane Potential in ZF4

**FIGURE 5:**
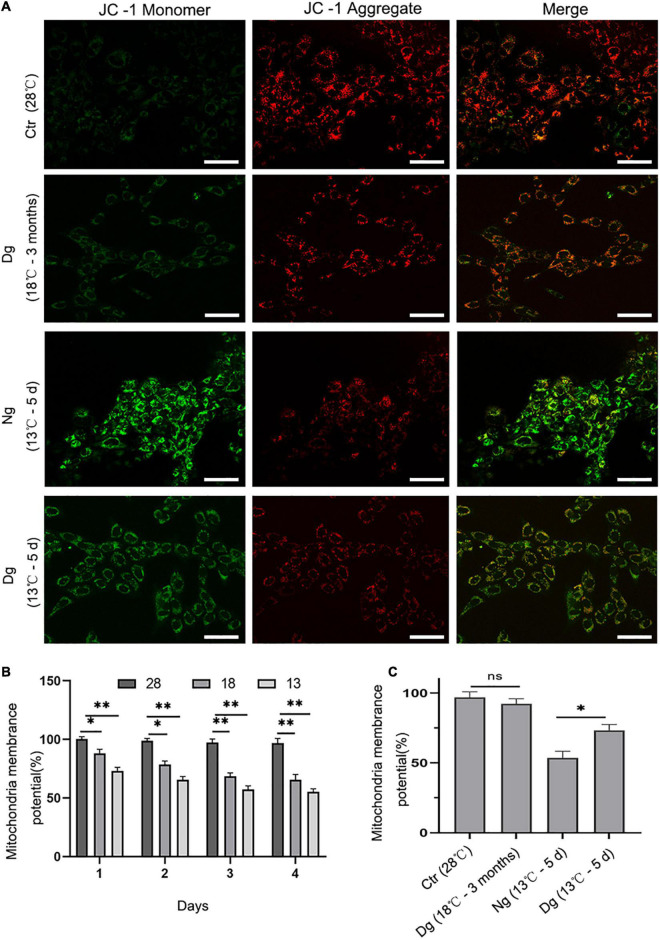
The effect of cold stress on mitochondrial membrane potential. Ctr, the control group; Dg, the domesticated group; Ng, the non-domesticated group. **(A)** Representative images were scanned by a confocal microscope after staining with JC-1. The red fluorescence indicates high mitochondrial membrane potential, while the green indicates low mitochondrial membrane potential. Scale bar = 50 μm. **(B,C)** Quantitative results of mitochondrial membrane potential by flow cytometry. Each experiment was performed in triplicate. The results are presented as the mean ± SD (*n* = 3); *P < 0.05, **P < 0.01.

Mitochondria have important roles in cellular aerobic respiration and oxidative phosphorylation, and their normal functioning is closely correlated with the level of intracellular ROS. JC-1 is a fluorescent probe for measuring mitochondrial membrane potential. When the mitochondrial membrane potential is normal, the JC-1 mitochondrial matrix aggregates to form polymers (J-aggregates) that emit red fluorescence. Conversely, when its mitochondrial membrane potential is low, JC-1 is a monomer in the mitochondrial matrix that produces green fluorescence. The shift of JC-1 from a red to green fluorescence state can be used to easily detect diminished cell membrane potential. Here, JC-1 staining and flow cytometry were both used to detect the changes in ZF4 cell mitochondrial membrane potential induced by low temperature stress. As evinced by Figure 5A, after JC-1 staining, the normal group cells showed red fluorescence, while the JC-1 in some cells changed from red fluorescence to green fluorescence under cold stress, indicating that mitochondrial membrane potential decreased under conditions of cold stress. Flow cytometry detected the mitochondrial membrane potential of ZF4 cells subjected to cold stress.

Compared with the control group (28°C), cold stress at 13 and 18°C for 4 days decreased the mean fluorescence intensity of the mitochondrial membrane potential by 55.28 and 65.51%, respectively (*P* < 0.01) (Figure 5B). In addition, the mitochondrial membrane potential of ZF4 cells decreased slightly after undergoing cold acclimation. Nevertheless, the differences in mitochondrial membrane potential between the cold acclimated group and control group were not pronounced (*P* > 0.05). Interestingly, when the acclimated group and the normal group were exposed to a lower temperature (13°C), statistically significant differences between these two groups were evident (*P* < 0.01). The mitochondrial membrane potential level was significantly lower in the normal group than the cold acclimated group (*P* < 0.05) (Figure 5C). Altogether, these results suggested that cold acclimation significantly relieved mitochondrial dysfunction in ZF4 cells.

### Cold Acclimation Reduces the Cold Stress-Induced Cellular Apoptosis of ZF4

**FIGURE 6:**
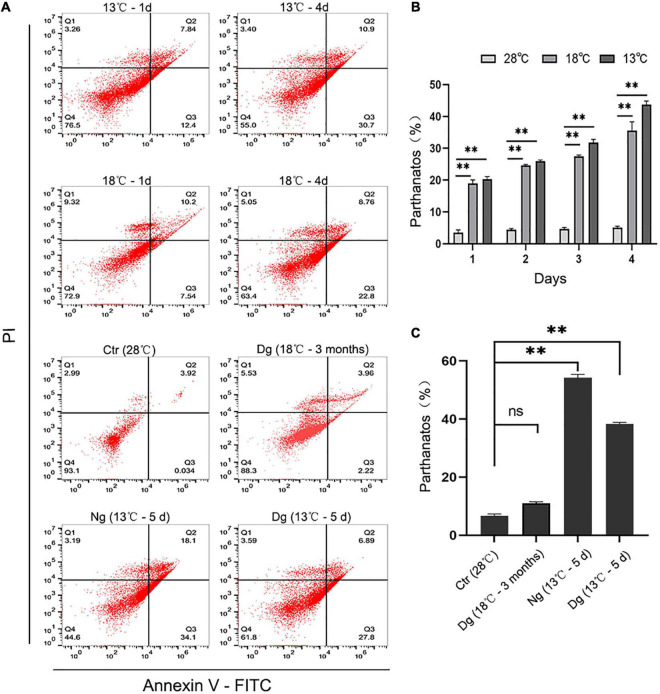
Cold acclimation alleviated cold-induced cell apoptosis in ZF4 cells. Ctr, the control group; Dg, the domesticated group; Ng, the non-domesticated group. **(A)** Analysis of cell apoptosis by flow cytometry. Apoptosis was evaluated with AnnexinV-FITC/PI staining. **(B,C)** Apoptotic cells were calculated and graphed according to the flow cytometry results in GraphPad Prism 8 software. The results are presented as the mean ± SD (*n* = 3); **P < 0.01.

It has been reported that increased ROS generation could lessen mitochondrial membrane potential (Δψm) and sequentially trigger mitochondria-dependent apoptosis. Therefore, we used Annexin V-FITC staining to detect the occurrence of ZF4 cell apoptosis induced by the cold stress. These results revealed the number of cell apoptosis increasing with a longer cold stress period relative to the control group. After 4 days of treatment at 13 and 18°C, the apoptosis rate of ZF4 cells was increased by 43.78 and 35.53%, respectively (both *P* < 0.01) (Figures 6A,B). However, the cold acclimation group only decreased the percentage of apoptosis by 10.97%. Similarly, we detected higher prevalence of apoptosis (54.24%) in the non-acclimation group than in the acclimation group (38.32%) after 5 days of cold treatment at 13°C (Figure 6C). These findings were consistent with the results of CCK-8 testing. Our results clearly showed that cold acclimation reduces the apoptosis induced by lower temperature stress.

## Discussion

As ectothermic animals, fish have particularly sensitive responses to changing temperatures in their aquatic environment. Over the past few decades, the major studies on the impact of climate change have focused on how high temperatures will affect fish biology. However, large-scale fish mortality and sublethal impacts caused by cold shock events also pose major challenges to fish survival (Viadero, 2005; O’Gorman et al., 2016). Generally, low temperature stress includes two aspects: cold acclimation and sudden temperature changes (Goos and Consten, 2002; Engelsma et al., 2003; Donaldson et al., 2008). Studies suggest that cold acclimation can improve the survival rate of fish under lethal low temperatures; for example, the survival rate of carp (*Cyprinus carpio*) at the lethal low temperatures of 8 and 10°C increased significantly after being cold-acclimated (Ge et al., 2020). Yet, our knowledge of the mechanisms through which low temperature domestication improves fish survival remains surprisingly limited. Therefore, here we studied zebrafish cells to explore the specific mechanism of cell survival after acute cold stress and cold acclimation. The present research contributes to a comprehensive understanding of cold tolerance in fish *via* novel insights into their cold acclimation-mediated physiology.

In this study, when facing acute cold stress, we found that cell viability decreased and the cytoskeleton shrank, whereas they remained normal after undergoing cold acclimation at 18°C for 3 months. These above findings indicate the successful establishment of a cold acclimation cell model. In addition, when the cells were exposed to a lower temperature (13°C), the cell viability of the acclimated group exceeded that of the non-acclimated group, indicating that the cells achieved cold tolerance ability and greater vigor after experiencing cold acclimation. Other studies have shown that acclimation generally fosters a corresponding compensation mechanism to maintain the homeostasis of the internal environment in fish (Goos and Consten, 2002; Engelsma et al., 2003). Also demonstrated in many animal studies, including those with zebrafish, is that cold acclimation affects the reduction in thickness of the compact myocardium and the changed collagen content when adjusting to a decrease in temperature (Johnson et al., 2014). In the process of cold acclimation, lipid catabolism and lipid oxidation were enhanced in milkfish (*Chanos chanos*) (Hsieh et al., 2003), and the actin cytoskeleton is known to be essential for maintaining tissue architecture (Egge et al., 2019). In the present study, we found that those cells lacking cold acclimation had a significantly lessened F-actin cytoskeleton structure after being transferred to a 13°C exposure, whereas the acclimated cells retained a robust cytoskeleton. This fits with other research that has shown the cytoskeleton plays a key role in maintaining the structure and functioning of cells at low temperatures (Colinet et al., 2017; Marteaux et al., 2018). Our study suggests acclimated cells attain better survival by improving their viability and modulating their cytoskeleton structures under cold stress conditions.

Cold stress can lead to an increase in endogenous ROS, such as hydroxyl radicals (-OH), superoxide free radicals (O_2_−), and hydrogen peroxide free radicals (H_2_O_2_). However, the cell has an intracellular antioxidant defense system, including SOD, CAT, and GSH, which play pivotal roles in scavenging ROS. When the production of ROS exceeds the body’s processing capacity, oxidative stress will occur (Lesser, 2006; An and Choi, 2010). In our study, antioxidant proteins and intracellular ROS were elevated at the onset of acute cold stress (13, 18°C). The upregulation of antioxidant enzymes protects the body from too much oxidative stress, and similar results have been reported in other fish studies (Cheng et al., 2017; Sánchez Nuño et al., 2018). Moreover, the level of mitochondrial membrane potential decrease and cell apoptosis increased with prolonged exposure to low temperature. Some researchers have pointed out that low temperature stress leads to the dysfunction of various biochemical reactions and physiological functions of fish. For example, acute cold stress affects the oxygen consumption rate and respiratory rate of fish (Maria Cristina and Paunescu, 2019), and a low temperature stress can alter the biochemical blood indexes and metabolism of large yellow croaker (*Pseudosciaena crocea*) (Liu et al., 2019). The intracellular ROS are mainly generated in mitochondria, and cold stress causes reduction the mitochondrial membrane potential and leads to enhanced ROS production (Morgan and Liu, 2011; Gerber et al., 2020; Xia et al., 2020). After a period of low temperature acclimation, the level of ROS and mitochondrial membrane potential in the cells returned to normal, and cell apoptosis decreased. This observation is consistent with previous findings that the apoptosis of zebrafish gill cells was decreased after undergoing cold acclimation (Chou et al., 2008).

Our study’s results suggest the production and clearance of ROS is in a state of dynamic balance during cold acclimation, which was able to lessen oxidative damage and decrease cell apoptosis. In fact, low concentrations of ROS promote cell proliferation, but high concentrations of ROS lead the body to form oxidative stress damage, DNA breaks, mutations, and peptide chain breaks (Engelsma et al., 2003; Tang et al., 2015). For the non-domesticated group, enzyme activity level and survival rates decreased, and the ROS level of the cells dramatically increased at 13°C for 5 days. In recent years, many reports have demonstrated that ROS plays an indispensable role in the signal transduction related to apoptosis (Tang et al., 2017; Wang et al., 2017; Feng et al., 2021). Hence, it can be inferred that the non-domesticated group cells cannot remove excess ROS, leading to an imbalance of oxidative stress and subsequently inducing cell apoptosis. After being transferred to a lower temperature (13°C), the cold acclimation group featured higher levels of antioxidant enzyme activity and a decreased ROS content. While the mitochondrial membrane potential decreased, the ZF4 cells’ rate of apoptosis rose in the non-domesticated group. Some reports have also suggested that low temperature acclimation improves mitochondrial function, including an increase in mitochondrial volume density and cristae surface area (Chung and Schulte, 2015; Yang et al., 2019). Accordingly, we could infer that cold acclimation results in enhanced mitochondrial function, which was able to reduce the accumulation of ROS and increase the mitochondrial membrane potential, and subsequently suppress the cell apoptosis caused by oxidative stress. In this way, the overall content of intracellular ROS would have been reduced, and mitochondrial functioning correspondingly changed to an enhanced state after domestication. These results explain why cold acclimation reduces oxidative stress to strengthen cold tolerance in ZF4 cells.

In conclusion, this study established a zebrafish ZF4 cell model for acute cold stress and long-term cold acclimation. It was determined that acute cold stress could induce oxidative stress in ZF4, and that low temperature acclimation could reduce oxidative damage and improve the low temperature tolerance of cells. These findings reveal the close connection between oxidative stress and the cold tolerance ability of fish, which should prove helpful for better understanding the cold tolerance mechanism of fish in general and finding ways to help them withstand low-temperature stress conditions in the future.

## Data Availability Statement

The raw data supporting the conclusions of this article will be made available by the authors, without undue reservation.

## Author Contributions

HW designed the experiment and wrote the manuscript. YW modified the original manuscript. MN and LH completed the data analysis. LC funding acquisition. All authors have read and agreed to the published version of the manuscript.

## Conflict of Interest

The authors declare that the research was conducted in the absence of any commercial or financial relationships that could be construed as a potential conflict of interest.

## Publisher’s Note

All claims expressed in this article are solely those of the authors and do not necessarily represent those of their affiliated organizations, or those of the publisher, the editors and the reviewers. Any product that may be evaluated in this article, or claim that may be made by its manufacturer, is not guaranteed or endorsed by the publisher.
